# Energy Efficiency Enhancement of Inductively Coupled Plasma Torch: Computational Study

**DOI:** 10.3390/ma15155213

**Published:** 2022-07-28

**Authors:** Samira Elaissi, Amira Ben Gouider Trabelsi, Fatemah H. Alkallas, Tahani A. Alrebdi, Kamel Charrada

**Affiliations:** 1 Department of Physics, College of Science, Princess Nourah Bint Abdulrahman University, P.O. Box 84428, Riyadh 11671, Saudi Arabia; 2 Research Unit of Ionized Backgrounds and Reagents Studies (UEMIR), Preparatory Institute for Engineering Studies of Monastir (IPEIM), University of Monastir, Kairouan Street, Monastir 5019, Tunisia

**Keywords:** performance analysis, energy efficiency, induction coupled plasma, computational fluid dynamics, flow pattern, temperature, parameter optimization, material processing

## Abstract

In this research, we studied the performance analysis of inductively coupled radiofrequency plasma “RF-ICP” torch used in multi-material processing. A 2D numerical model built with COMSOL Multiphysics was used to study the discharge behavior and evaluate the overall efficiency transmitted into the plasma system. The temperature and velocity flow of the plasma were investigated. The numerical results are consistent with previous experimental studies. The temperature and velocity profiles are represented under a wide range of RF power and for different sheath gas flow rates. With increasing power, the radial peak temperature typically shifts towards the wall. The resistance of the torch rises whereas the inductance diminishes with increasing RF power. The overall dependency of the coupling efficiency to the RF power is also estimated. The stabilization of the plasma flow dependency to the sheath swirl flow was investigated. The incorporation of Helium (0.02%) into an Argon gas was established to minimize the energy lost in the sidewall. The number and spacing of induction coil numbers affects the temperature and flow field distribution. A valuable approach to designing and optimizing the induction plasma system is presented in the proposed study. The obtained results are fundamental to specify ICP torch design criteria needed for multi-material processing.

## 1. Introduction

For the last two decades, considerable interest has been devoted to applying inductively coupled radio frequency plasma (RF-ICP) in multi-material processing due to its higher temperature and plasma density, as well as the absence of contaminating electrodes [1,2]. Hence, the ICP thermal plasma torch has rapidly replaced flames, direct current plasma, and other sources of ionization/excitation in various industrial fields, including semiconductor, biomedical, clinical, environmental, and geological or geochemical [3,4,5].

The distinguishable performance of ICP compared to other plasma generation techniques have been widely required during the production of high purity materials, thin deposition [6,7], surface modification [8,9] and waste treatment [10,11,12], and chemical synthesis and chemical processing [13,14,15]. For material processing in particular, ICP thermal plasma covers numerous applications such as spectrochemical analysis, powder spheroidization, etching, surface treatment, sintering, spray coating, and material synthesis including nanoparticles, composites, and catalysts [16,17,18,19].

A commercially successful ICP technology requires the minimization of energy losses and the optimization of the process parameter affecting the plasma appearance and morphology [20]. Indeed, the estimation of the plasma resistance and plasma impedance under different operating conditions is important to design a radiofrequency (RF) generator. Moreover, a proper estimation of the plasma heated area must be determined to reduce the energy lost close to the sidewall. Therefore, to successfully implement an experimental RF-ICP system, it will be crucial to have a profound understanding of its basic functionalization and control its discharge conditions in detail [21].

Meanwhile, plasma is a multifunctional fluid characterized by high chemical reactivity, energy density, and variable transport parameters. Furthermore, RF induction discharge remains inherently unstable while applying a magnetic field making the plasma control difficult. Hence, the experimental measurements inside the torch are very difficult to perform due to the extremely high local temperature. A computational study is therefore indispensable to ensure a better comprehension of the ICP’s chemical and physical properties and to understand the strong coupling between the Maxwell equation, momentum, and energy [22]. This will develop an excellent tool for diagnosing industrial system problems, determining the plasma impedance, analyzing power dissipated in the ICP system, and avoiding costly and time-consuming experimental processes [23].

Several experimental and computational models studying ICP discharge exist so far. Boulos was the first to develop a thermal fluid electromagnetic thermo-fluid model for thermal plasma induction in which he determined the thermal field and gas flow inside the plasma [24]. In their study, Punjabi et al. simulated the ICP torch temperature distribution under a variety of conditions and with high-frequency coil positions to study the heat transfer using the computational fluid dynamics (CFD) method [25]. Linder and Bogaerts introduced an ICP model at atmospheric pressure where they analyzed the great influence on the ICP center due to Helium addition in pure Argon gas causing elongation of the center channel [26]. A magneto-dynamic study of induction plasma discharges and their interaction with power sources has been simulated using an integrated model [27,28]. A new 2D model given by Xue et al. demonstrates that the coil angle changes highly influence the flow field as well as the temperature distribution [29]. Later, a comparison of the He-ICP discharge features at atmospheric pressure with those of Ar-ICP has been presented by Cai et al. [30]. Bernardi et al. developed a method for studying the ICP electromagnetic field distribution based on the comparison of three different techniques using Ansys Fluent [31]. A boundary element finite difference method is introduced by Fouladgar and Chentouf to estimate the inductance and resistance dependency to temperature in RF-ICP torches [32]. Another mathematical simulation model of the temperature and flow distribution is carried out later by Ye et al. in an RF-ICP torch to elucidate the basics of turbulence phenomena and study the associated heat transfer effect [33].

Herein, the energy efficiency enhancement of the atmospheric pressure ICP torch was investigated. A two-dimensional “2D” axisymmetric model of inductively plasma discharge is presented through COMSOL Multiphysics [34]. First, a comparison of our numerical results with previous experimental results is presented to demonstrate the accuracy of our model simulation. Second, the temperature and velocity flow distribution inside the ICP torch are analyzed. Then, a parametric study was carried out to identify which factors affect the temperature and flow distribution. The plasma resistance is calculated based on a variety of control parameters including radiofrequency power and gas flow rate. The optimum conditions to minimize the energy loss close to the sidewall were determined. Efficient material processing requires successfully implementing of RF-ICP in practice.

## 2. Torch Geometry and Operating Conditions

**Figure 1:**
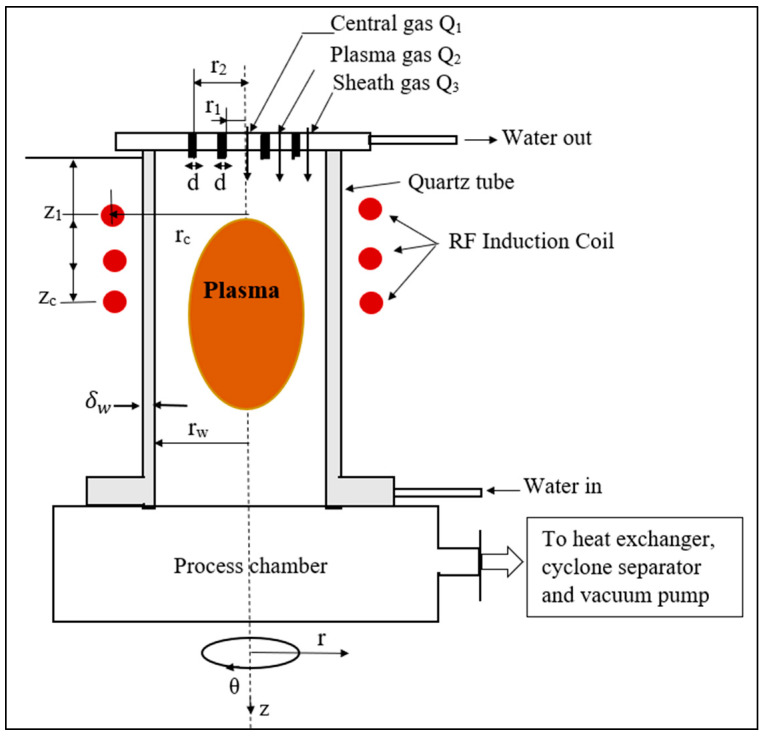
Representation of RF-ICP torch.

Figure 1 illustrates a simplified presentation of radiofrequency inductive thermal plasma (ITP) system. Three nozzles are located on the gas inlet for central, plasma, and sheath gases, respectively [35]. RF induction coils (3 MHz, 15 kW), produce an RF-ICP discharge and maintain it inside the torch.

Table 1 summarizes the operational conditions, and the torch geometry dimensions regarding an industrial RF-ICP torch [36].

## 3. Model Description

### 3.1. Basic Model Assumptions

The principal assumptions used in the developed model are as follows [37]:

(i). The plasma system is modeled by a two-dimensional axisymmetric configuration, and the outer inductor is represented by a series of parallel conductive rings infinitely thin. The significant voltage that appears in windings, acting as an axial electric field inducing a dielectric barrier discharge type, can be avoided when placing the torch vertically rather than horizontally.
(ii). The flow of working fluid is at a steady state, compressible, with a small Mach number (Ma < 0.3).
(iii). During torch operation, we consider moderate mass flow rates with a low Reynolds number (Re ~ 500) and laminar flow.
(iv). The plasma is in a state of local thermodynamic equilibrium (LTE).
(v). Optically thin plasma, so radiation reabsorption is negligible.
(vi). Plasma displacement current can be ignored as it is relatively small compared to conductive current.
(vii). The heat generated by viscous dissipation is neglected in the energy equation.
(viii). Ohmic heating is responsible for volumetric power input.

### 3.2. Governing Equations and Boundary Conditions

Based on the previous assumptions and in two-dimensional axisymmetric cylindrical coordinates, the governing equation could be written as: [38]

Continuity:

(1) $$\frac{\partial \left(\rho u\right)}{\partial z}+\frac{1}{r}\frac{\partial \left(r\rho v\right)}{\partial r}=0$$

Axial Momentum

(2) $$\frac{\partial \left(u\rho u\right)}{\partial z}+\frac{1}{r}\frac{\partial \left(\mathrm{rv}\rho u\right)}{\partial r}=-\frac{\partial p}{\partial z}+2\frac{\partial }{\partial z}\left(\mu \frac{\partial u}{\partial z}\right)+\frac{1}{r}\frac{\partial }{\partial r}\left\{\mu r\left(\frac{\partial u}{\partial r}+\frac{\partial v}{\partial z}\right)\right\}+F_{z}$$

Radial Momentum

(3) $$\frac{\partial \left(u\rho v\right)}{\partial z}+\frac{1}{r}\frac{\partial \left(\mathrm{rv}\rho v\right)}{\partial r}=-\frac{\partial p}{\partial r}+\frac{\partial }{\partial z}\left\{\mu \left(\frac{\partial u}{\partial z}+\frac{\partial v}{\partial r}\right)\right\}+\frac{2}{r}\frac{\partial }{\partial r}\left(\mu r\frac{\partial v}{\partial r}\right)-2\mu \frac{v}{r^{2}}+\rho \frac{w^{2}}{r}+F_{r}$$

Swirl momentum

(4) $$\frac{\partial \left(u\rho w\right)}{\partial z}+\frac{1}{r}\frac{\partial \left(\mathrm{rv}\rho w\right)}{\partial r}=\frac{\partial }{\partial z}\left\{\mu \left(\frac{\partial w}{\partial z}\right)\right\}+\frac{1}{r}\frac{\partial }{\partial r}\left(\mu r\frac{\partial w}{\partial r}\right)-\frac{w}{r}\left(\rho v+\frac{\partial \mu }{\partial r}+\frac{\mu }{r}\right)$$

Enthalpy

(5) $$\frac{\partial \left(u\rho h\right)}{\partial z}+\frac{1}{r}\frac{\partial \left(\mathrm{rv}\rho h\right)}{\partial r}=\frac{\partial }{\partial z}\left\{\frac{\kappa }{C_{p}}\left(\frac{\partial h}{\partial z}\right)\right\}+\frac{1}{r}\frac{\partial }{\partial r}\left(r\frac{\kappa }{C_{p}}\frac{\partial h}{\partial r}\right)+Q_{P}-Q_{\mathrm{rad}}$$

where r and z are the distance in radial and axial direction, V, u, and w are the radial, axial and azimuthal velocities, respectively. P is the pressure, h is the enthalpy, Q_R_ is the volumetric radiation heat losses, and Q_P_ is the local energy dissipation rate. µ, ρ, κ, and C_p_ are the viscosity, density, thermal conductivity, and specific heat at constant pressure, respectively.

Maxwell’s equations govern the electromagnetic field as follows:

(6) ∇·E=0

(7) ∇·H=0 

(8) $$\nabla \times E=-\mu_{0}\frac{\partial H}{\partial t}\;$$

(9) ∇×H=J 

Here, **E** and **H** are the electric field vector and the magnetic field vector, respectively. µ_0_ = 4π × 10^−7^ H m^−1^ represents the free space permeability and **J** denotes the total current density.

The intensity of the magnetic field is given as

(10) $$\mu_{0}H=\nabla \times A$$

where **A** is the magnetic vector potential.

Replacing Equation (10) into Equation (9)

(11) $$\nabla \times \left(\frac{\nabla \times A}{\mu_{0}}\right)=J$$

and using the relation $\nabla \times \nabla \times A=\nabla \left(\nabla \cdot A\right)-\nabla^{2}A$ and ∇·A=0, we get

(12) $$\nabla^{2}A=-\mu_{0}J\;$$

Here, the total current density **J** is divided into two components, including the current density created by the applied voltage at the coil that ends **J_coil_**, as well as the current density developed by the induced electric field in the plasma and the coil **J_ind_**.

(13) $$\nabla^{2}A=-\mu_{0}\left(J_{coil}+J_{ind}\right)$$

Assuming that the coil is consisting of parallel rings, the vector potential and the electric field have only tangential components.

(14) $$A=\left(0,{\;A}_{\theta },\;0\right)$$

Using Equation (14), Equation (13) can be given as

(15) $$\nabla^{2}A_{\theta }-A_{\theta }/r^{2}=-\mu_{0}\left(J_{coil}+J_{ind}\right)$$

The θ component of the vector potential must consider real and imaginary additional scalars:

(16) $$A_{\theta }=A_{\theta R}+{\mathrm{iA}}_{\theta I}$$

where A_θR_ is the real component of the vector potential, $A_{\theta }$ and A_θI_ is the imaginary component.

The electromagnetic coupling equations are written as follows:

(17) $$\frac{1}{r}\frac{\partial }{\partial r}\left(r\frac{\partial A_{\theta R}}{\partial r}\right)+\frac{\partial^{2}A_{\theta R}}{\partial z^{2}}-A_{\theta R}/r^{2}{=-\mu }_{0}{(J}_{\mathrm{coil}}{+J}_{\mathrm{ind}})$$

(18) $$\frac{1}{r}\frac{\partial }{\partial r}\left(r\frac{\partial A_{\theta I}}{\partial r}\right)+\frac{\partial^{2}A_{\theta I}}{\partial z^{2}}-A_{\theta I}/r^{2}{=-\mu }_{0}{(J}_{\mathrm{coil}}{+J}_{\mathrm{ind}})$$

In the plasma region:

$J_{ind}=\sigma E=-{i\omega \sigma A}_{\theta }$ and $J_{coil}=0$, so that

(19) $$\nabla^{2}A_{\theta R}-(1/r^{2}+{i\omega \mu }_{0}\sigma )A_{\theta R}=0$$

(20) $$\nabla^{2}A_{\theta I}-(1/r^{2}+{i\omega \mu }_{0}\sigma )A_{\theta I}=0$$

where, σ denotes the electrical conductivity, ω (= 2 πf) represents the angular frequency, and f is the induction current frequency.

In the coils:

$J_{ind}=0$ and $J_{coil}=I_{\mathrm{coil}}/S_{\mathrm{coil}}$ (where $I_{\mathrm{coil}}$ and $S_{\mathrm{coil}}$ are the coil current and the coil cross-section, respectively)

(21) $$\nabla^{2}A_{\theta R}-A_{\theta R}/r^{2}=-\mu_{0}J_{coil}$$

(22) $$\nabla^{2}A_{\theta I}-A_{\theta I}/r^{2}=0$$

Anywhere else, there is no current source, so that

(23) $$\nabla^{2}A_{\theta R}-A_{\theta R}/r^{2}=0$$

$$\nabla^{2}A_{\theta I}-A_{\theta I}/r^{2}=0$$

The electric field can be obtained from the Maxwell equations by substitution of Equation (10) into Equation (8)

(24) $$\nabla \times E=-\mu_{0}\frac{\partial \left(\frac{\nabla \times A}{\mu_{0}}\right)}{\partial t}$$

The scalar potential is zero in the absence of an electrostatic field. So, the intensity of the electric field is calculated as:

(25) $$E=-\frac{\partial A}{\partial t}$$

As a result, azimuthal electric field intensity E_θ_, radial magnetic field component H_z,_ and axial magnetic field component H_r_ are calculated as follows:

(26) $$E_{\theta }=-{i\omega A}_{\theta }\;\mathrm{where}\;i^{2}\;=-1\;$$

(27) $$\mu_{0}H_{z}=\frac{1}{r}\frac{\partial }{\partial r}\left({\mathrm{rA}}_{\theta }\right)$$

(28) $$\mu_{0}H_{r}=-\frac{\partial }{\partial r}A_{\theta }$$

If we apply the Lorentz force to the momentum equations, we get:

(29) FLz=−12μ0σ Re[EθHr*]

(30) FLr=12μ0σ Re[EθHz*]

where Re[z] and z^*^ represent the real part and the conjugate part of the complex number z, respectively.

The conversion efficiency can be given as

(31) $$\eta_{c}=\frac{P_{\mathrm{diss}}}{P_{\mathrm{coil}}}\times 100$$

P_diss_ is the RF dissipated power in plasma and P_coil_ is the power supplied to the coil, calculated as [39]:

(32) $$P_{\mathrm{diss}}=\int_{V_{d}}^{\;}Q_{P}{\mathrm{dV}}_{d}$$

(33) $$P_{\mathrm{coil}}=\frac{1}{2}{\pi f\mu }_{0}\int_{V_{d}}^{\;}{(H}_{r}^{2}{+H}_{z}^{2}{)\mathrm{dV}}_{d}$$

where V_d_ is total discharge volume and Q_P_ is the local energy dissipation rate, given as:

(34) $$Q_{P}=\frac{1}{2}\sigma \left[E_{\theta }E_{\theta }^{*}\right]$$

According to the ICP conservation equations, the boundary conditions are defined as [40]:

For the inlet conditions (z = 0):

$$u=\{\begin{array}{c} Q_{1}/{\pi r}_{1}^{2},\;r<r_{1}\\ 0,{\;r}_{1}\leq r\leq r_{1}+d\\ Q_{2}/\pi \left(r_{2}^{2}-r_{1}^{2}\right),\;r_{1}+d\leq r\leq r_{2}\\ 0,\;r_{2}\leq r\leq r_{2}+d\\ Q_{3}/\pi (r_{w}^{2}-r_{2}^{2}),\;r_{2}+d\leq r\leq r_{w}\\ \; \end{array}$$

v=0

$$w=v_{\theta }r_{w}$$

T = 300 K

$$\frac{\partial A_{\theta R}}{\partial z}=\frac{\partial A_{\theta I}}{\partial z}=0$$

where Q_1_, Q_2_, and Q_3_ are central gas, plasma gas, and sheath gas flow rates, respectively. r_1_, d, r_2_, r_c_ and r_w_ are the radius of the injection tube, the tube thickness, the radius of the intermediate tube, the radius of the coil, and the radius of the confinement tube, respectively. v_θ_ indicates the swirl angular velocity.

For the torch axis (r = 0):

$$\frac{\partial u}{\partial r}=v=w=\frac{\partial h}{\partial r}=A_{\theta R}=A_{\theta I}=0$$

For the torch wall (r = r_w_):

u=v=w=0

$$\kappa \frac{\partial T}{\partial r}=\frac{\kappa_{c}}{\delta_{w}}\left(T_{s}-T_{w}\right)$$

where, κ_c_ is the quartz wall thermal conductivity (κ_c_ = 1.047 W/m K), and δ_w_ represents the thickness of the tube wall. T_w_ and T_s_ are the temperature of the external surface of the tube (T_w_ = 300 K) and the temperature of the inside the surface of the tube, respectively.

The real part A_θR_ and the imaginary part A_θI_ of the vector potential A_θ_ can be expressed as:

(35) $$A_{\theta R}=\frac{\mu_{0}I}{2\pi }\sqrt{\frac{r_{c}}{r_{w}}}\sum_{i}^{\mathrm{coil}}G\left(k_{i}\right)+\frac{\mu_{0}\omega }{2\pi }\sum_{m}^{C.V}\sqrt{\frac{r_{p}}{r_{w}}}\sigma_{p}A_{\theta I,p}s_{p}G\left(k_{p}\right)$$

The first summation is over the number of the coils and the second one covers the current carried over the discharge region.

(36) $$A_{\theta I}=-\frac{\mu_{0}\omega }{2\pi }\sum_{m}^{C.V}\sqrt{\frac{r_{p}}{r_{w}}}\sigma_{p}A_{\theta R,p}s_{p}G\left(k_{p}\right)$$

Considering:

$$G\left(k\right)=\frac{\left(2-k^{2}\right)K\left(k\right)-2E\left(k\right)}{k}$$

$$k_{p}^{2}=\frac{4r_{w}r_{p}}{{\left(r_{p}+r_{w}\right)}^{2}+{\left(z_{b}-z_{p}\right)}^{2}},{\;k}_{i}^{2}=\frac{4r_{i}r_{w}}{{\left(r_{i}+r_{w}\right)}^{2}+{\left(z_{i}-z_{b}\right)}^{2}}$$

Here, s_p_, r_p_, and σ_p_ are the cross-section, radius, and electrical conductivity of the m^th^ control volume. z_i_ and r_i_ are the height and the radius of the i^th^ coil and z_b_ is the boundary height. K(k) and E(k) are the first and the second kind of complete elliptic integrals, respectively, and they are used to evaluate the magnetic vector potential at wall boundary. The numerical calculation of these integrals is given in [41].

For the exit of the torch

$$\frac{\partial \left(\rho u\right)}{\partial z}=\frac{\partial v}{\partial z}=\frac{\partial w}{\partial z}=\frac{\partial h}{\partial z}=\frac{\partial A_{\theta R}}{\partial z}=\frac{\partial A_{\theta I}}{\partial z}=0$$

Based on azimuthal symmetry distribution, the plasma can be described as a collection of eddy currents that are magnetically coupled to the induction coil. Hence, the coil voltage can be determined as:

(37) $$V_{\mathrm{coil}}=\overset{\mathrm{coil}}{\underset{i}{\sum }}U_{i}=\overset{\mathrm{coil}}{\underset{i}{\sum }}2{\pi r}_{i}\left(\frac{I_{\mathrm{coil}}}{\sigma_{\mathrm{coil}}S_{\mathrm{coil}}}+{i\omega A}_{\theta i}\right)\;$$

The complex impedance of the torch can be obtained by combining Equation (37) and Equations (35) and (36) and calculating the rate of the coil voltage to the coil current [42]:

$$\left|Z_{T}\right|=\frac{V_{\mathrm{coil}}}{I_{\mathrm{coil}}}=\left|R_{\mathrm{torch}}+{\mathrm{iX}}_{\mathrm{torch}}\right|$$

where R_torch_ is the torch resistance and X_torch_ is the torch reactance.

Finally, the torch resistance and torch inductance are performed as follows [43]:

$$R_{\mathrm{torch}}=R_{\mathrm{coil}}+R_{\mathrm{plasma}\;}$$

(38) $$R_{\mathrm{torch}}=\sum_{i=1}^{\mathrm{coil}}\left(\frac{2{\pi r}_{i}}{\sigma_{\mathrm{coil}}S_{\mathrm{coil}}}+\frac{\omega^{2}\mu_{0}^{2}}{2\pi }\times \sum_{m=1}^{C.V.}r_{i}\sigma {}_{p\;}A_{\theta R,p}^{*}s_{p}\sqrt{\frac{r_{p}}{R_{i}}}G\left(k_{i,p}\right)\right)$$

In Equation (38), the first term represents the coil ohmic resistance (R_coil_), and the second term denotes the plasma resistance of the plasma (R_plasma_). 

The torch inductance can be calculated as:

$$L_{\mathrm{torch}}=\frac{X_{\mathrm{torch}}}{\omega }$$

And we obtain:

$$L_{\mathrm{torch}}=L_{\mathrm{coil}}-{\;L}_{\mathrm{plasma}\;}$$

(39) $$L_{\mathrm{torch}}=\sum_{i=1}^{\mathrm{coil}}\left(\sum_{n=1}^{\mathrm{coil}}\Lambda_{n}-\frac{{\omega \mu }_{0}^{2}}{2\pi }\times \sum_{m=1}^{C.V.}r_{i}\sigma_{p}A_{\theta I,p}^{*}s_{p}\sqrt{\frac{r_{p}}{R_{i}}}G\left(k_{i,p}\right)\right)\;$$

In Equation (39), the first term indicates mutual inductance of single-coil turn (i) with the other turns of the coil, and the second one denotes mutual inductance between the plasma and coil turn (i).

Where $\Lambda_{n}=\{\begin{array}{c} r_{i}\mu_{0}\sqrt{\frac{r_{n}}{r_{i}}}G\left(k_{i,n}\right),\;i\neq n\\ N^{2}r_{i}F_{0}\times 1\times 10^{-9},\;i=n \end{array}$

And $A_{\theta }{}^{*}=2\pi \frac{A_{\theta }}{\mu_{0}I_{\mathrm{coil}}}$

F_0_ is the shape factor in Grover’s self-inductance formula [44] and N is the number of coil turns.

When i = n, the first term becomes the impedance due to the self-inductance, and Grove’s self-inductance formula was utilized rather than Maxwell’s mutual inductance formulation.

### 3.3. Thermodynamic and Transport Properties

At high pressures, the plasma is considered in local thermodynamic equilibrium (LTE), and the demixing of chemical elements can be neglected. So, the thermodynamic and transport properties can be expressed as a function of temperature and pressure only.

The thermodynamics plasma properties including viscosity μ, specific enthalpy h, thermal conductivity κ, mass density ρ, specific heat C_p_, electrical conductivity σ for atmospheric pressure Argon as a function of temperature and pressure, are obtained from reference [45].

In this case, the distribution function of thermal plasma constituents is Maxwellian [46]. For pure Argon, the radiative loss term is calculated according to the relation given by Bernardi et al. [47]:

(40) $$Q_{\mathrm{rad}}=5600\left(T-9500\right)+181{\left(T-9500\right)}^{2}$$

where T is the temperature (T > 9500).

### 3.4. Calculation Conditions

The computational model was simulated using the COMSOL Multiphysics software 5.4. Three physics modules were implemented in the model, including fluid dynamics, heat transfer electric, and the magnetic field. Maxwell equations are solved by the magnetic field module to determine the electromagnetic fields generated by an alternating current within the coil. A 2D axisymmetric cylindrical geometry of the torch is incorporated into the model, simplifying the mathematical simulation of the torch. The finite element method was performed to resolve the governing equations using nonuniform triangular meshes [48]. 

**Figure 2:**
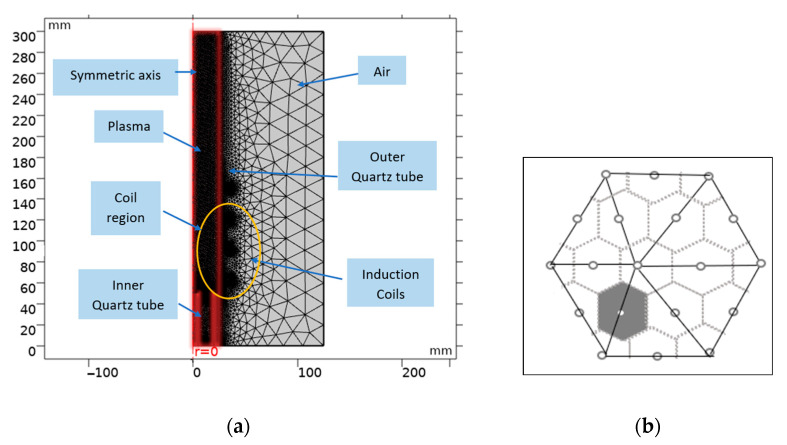
Computational domain of RF-ICP torch (**a**) and the intersection of finite element and control volumes (shaded areas) (**b**).

To assure the accuracy of the calculation, the calculation domain was covered with a minimum mesh size of 0.019 mm, a maximum mesh size of 0.09 mm, and a growth rate of 1.08. An independent grid study determined that a resolution of around 48 cells/mm was optimal. An intel core i7-HP computer with 16 GB of RAM was used to run the simulation for two to four hours. The details of the geometry and grid distribution have been shown in Figure 2. Control volume and finite element formulation are combined in the numerical framework [49]. Figure 2b shows the two-dimensional boundaries of the control volume.

## 4. Results and Discussion

### 4.1. Comparison of Numerical and Experimental Plasma Characteristics

**Figure 3:**
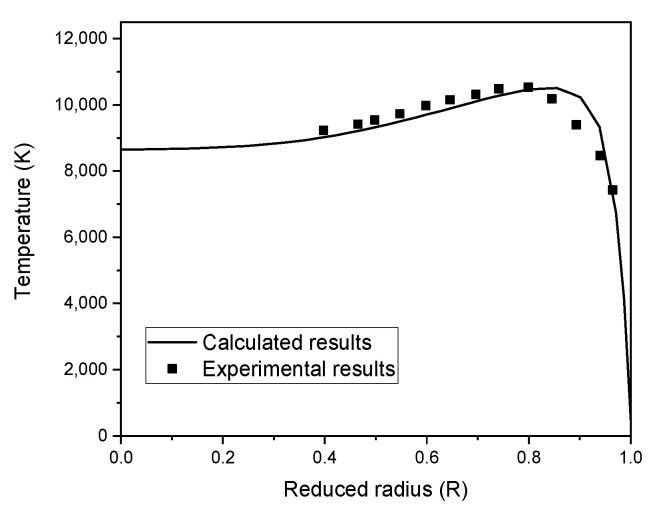
Comparison of calculated radial temperature profiles with experimental data of Punjabi et al. [50] in the centerline of the coil region (z = 192 mm).

Initially, we proceed by correlating numerical results and experimental measurements obtained by Punjabi et al. [50] using optical emission spectroscopy under the same operational conditions. In Figure 3, the radial temperature profile is represented in the region of the coil’s centerline for plasma (z = 192 mm) at 7.5 kW with a 10 lpm sheath gas flow rate and a 60 mm diameter tube. Numerical and experimental results are in good accordance with a minor over-prediction of around 5% near the plasma edge.

**Figure 4:**
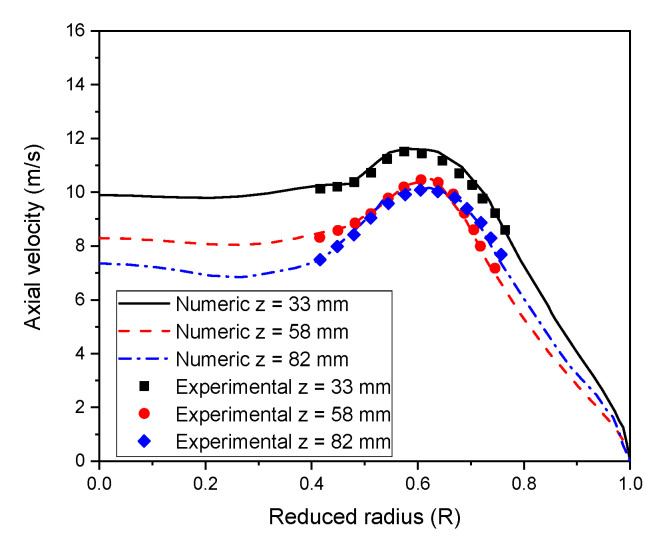
Comparison of calculated axial velocity profiles with experimental data of Lesinski et al. [51] for various axial locations (z = 33, 58, and 82 mm).

In the same way, we investigated the variation of the numerical axial velocity profile using a 50 mm diameter tube, 4.6 kW plasma power, and 63 lpm sheath gas rate (see, Figure 4). Numerical results and experimental data obtained by Lesinski et al. [51] using laser doppler anemometry seem to agree well with each other at all the axial locations (z = 33, 58, and 82 mm).

### 4.2. Analysis of Temperature and Velocity Flow Distributions in ICP Torch

An inductively coupled plasma torch operated in the same condition as Punjabi et al. [52] using Argon gas under atmospheric pressure is computationally analyzed. The flow rate at central Q_1_, plasma Q_2_, and sheath Q_3_, is equal to 1 lpm, 3 lpm, and 21 lpm, respectively. Sheath gas flow is introduced under a swirl flow condition, where swirl angular velocity is 800 rad/s. The oscillation’s frequency is 3 MHz, and the discharge power is maintained at 15 kW.

**Figure 5:**
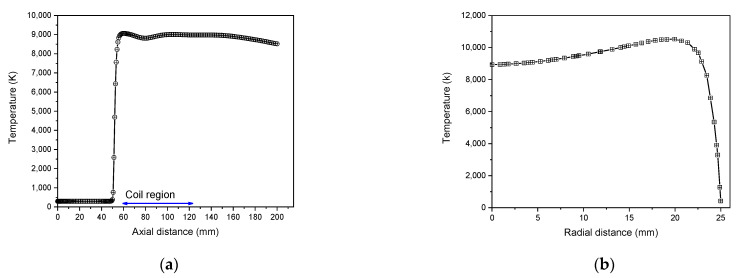
Axial temperature (r = 0) (**a**) and radial temperature (z = 92 mm) (**b**) distribution in ICP torch.

As shown in Figure 5a, the axial temperature profile in the symmetry axis (r = 0), indicates that plasma begins heating up as soon as it passes the coil’s first loop. Gradually, the temperature rises until it reaches saturation. 

In Figure 5b, the radial distribution of temperature represented at the second coil position (z = 92 mm) shows that near the central axis, the temperature is greater than 8000 K and becomes the highest up at 20 mm from the axis. At the quartz wall, a cold boundary layer is established, where the temperature is less than about 600 K [53].

**Figure 6:**
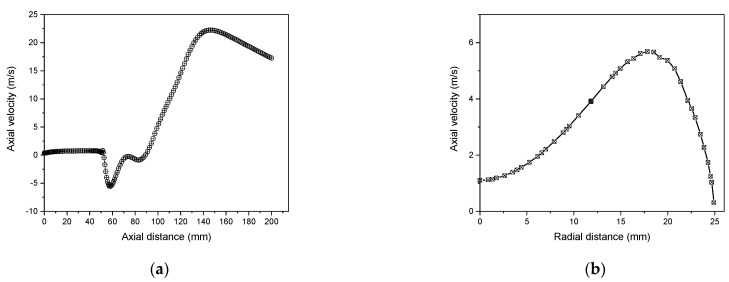
Axial temperature (r = 0) (**a**) and radial distribution of axial velocity (z = 92 mm) (**b**) profiles in ICP torch.

The axial velocity profile along the torch axis represented in Figure 6a shows that the velocity profile increases and then drops slowly close to the torch exit. Negative velocity values resulting in significant circulating flow in pure Argon within the torch can be attributed to energy dissipation. The distribution of axial velocity in the radial direction in the region of the coil’s centerline (z = 92 mm) is illustrated in Figure 6b. The axial velocity progressively rises until attending the wall associated with the elevation of the temperature, where it reaches its maximum [54].

### 4.3. Variation of Plasma Parameters

To enhance the torch performance, the most effective and efficient process would be to control the geometrical and operational parameters. Therefore, we investigated the plasma characteristics dependency on the RF power changes between 3 and 15 kW and the sheath gas flow rate variation in the range from 5 to 31 lpm. This is to optimize the parameters model, improving torch efficiency in material processing [55]. 

#### 4.3.1. Effect of RF Power

**Figure 7:**
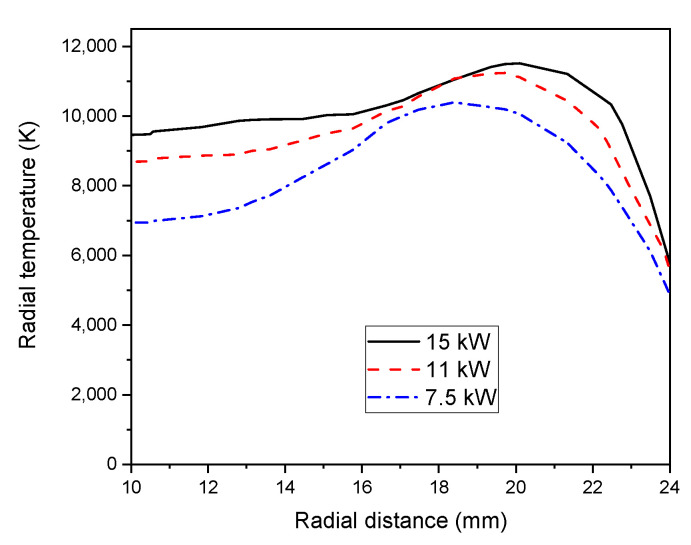
Radial Temperature field (z = 92 mm) with different RF power (7.5, 11, and 15 kW) with a sheath gas flow rate of 21 lpm (zoomed view from 10 to 24 mm).

Figure 7 shows a zoomed view of the radial temperature profiles (from 10 to 24 mm) for varied RF input powers (P = 7.5, 11, and 15 kW) at the same axial position z = 92 mm. The elevation of input power raises the temperature, then, the plasma core expands along the axial and radial direction due to the increase in the plasma core temperature. Herein, the plasma core moves away from the centerline towards the wall, while reducing the skin depth. 

Consequently, the plasma electric conductivity becomes important own to the higher ionization. As a result of convective heat transfer, the temperature progressively diminishes, far off the peak value, and electrical conductivity drops. On other hand, the plasma temperature illustrates a decrease in its value while decreasing the plasma power [56].

**Figure 8:**
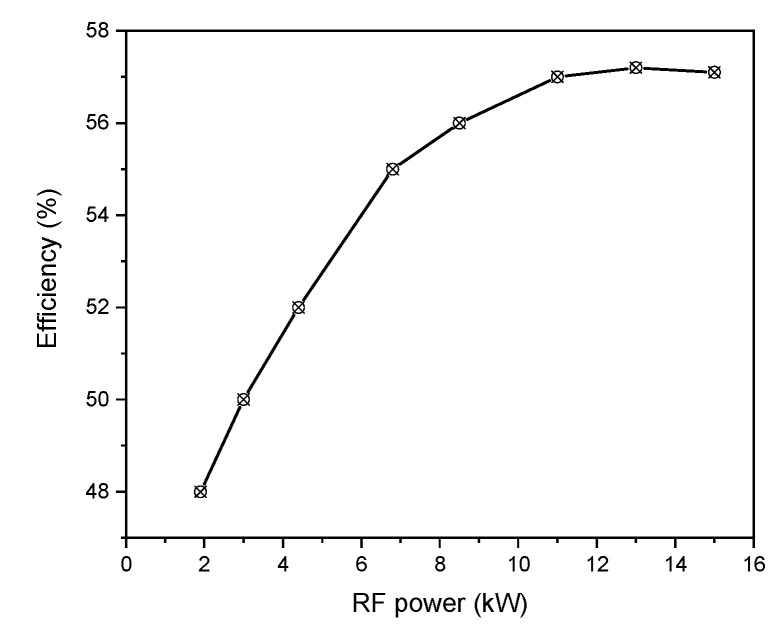
Torch efficiency versus RF power.

The efficiency of plasma torch dependency on the RF power is represented in Figure 8. By increasing the injection power, the coupling efficiency is enhanced resulting in a higher discharge volume and therefore a corresponding increase in the magnetic flux linked cross-section. 

Indeed, by dissipating the power in the plasma and the inductor resistance, the torch efficiency depending on their ratio is enhanced [57].

**Figure 9:**
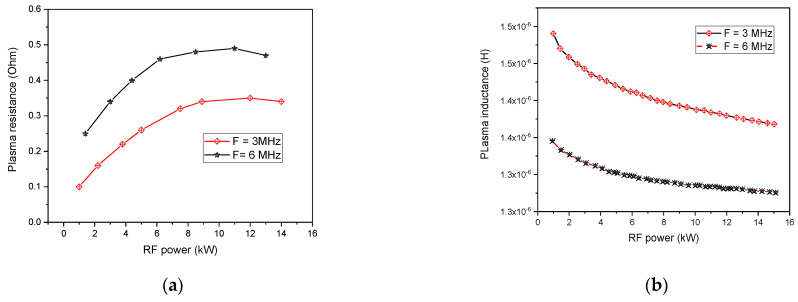
Plasma resistance (**a**) and plasma inductance (**b**) with the variation of RF power.

On other hand, the resistance and inductance variation with RF power are shown in Figure 9. At high power, the resistance rises with a simultaneous increase in the temperature (see, Figure 7). Although, the rise in temperature is commonly accompanied by a reduction in resistance. Such an adverse effect is imaged through the asymptotic behavior located in the resistance variation as a function of RF power (see, Figure 9a). 

On the other hand, Plasma inductance is one of the plasma characteristics highly dependent on the coil’s magnetic flux association. In Figure 9b, the inductance slowly decreases with increasing RF power. Indeed, the temperature increases with rising RF power that simultaneously expands plasma core volume. This allows a higher magnetic flux penetration into the plasma core, reducing the separation distance between the plasma and the coil, which is accountable for flux leakage.

Furthermore, we studied the plasma resistance and inductance variation with the frequency changes. A higher value is found for the plasma resistance while increasing the plasma frequency (see, Figure 9a). Indeed, with elevated frequency, the Joule heating area approaches the wall with a thinner accentuated electrical conductivity region and a higher temperature zone closer to the wall. This is due to the skin effect linked to skin depth, defined as the tendency for alternating current (AC) signals to flow near the outer edge of the electrical conductor [58]:

(41) $$\delta ={\left({\pi \mu }_{0}\sigma f\right)}^{-\frac{1}{2}}$$

The Lorentz force also concentrates near the wall, and then the vortices near the coil region are vanished.

Contrary, the plasma inductance reduces more with rising frequencies (see, Figure 9b). This affects torch efficiency, which is related to the ratio of dissipated power to coil power. Therefore, a high plasma frequency ensures a better torch efficiency.

#### 4.3.2. Influence of Sheath Gas Flow Rate

**Figure 10:**
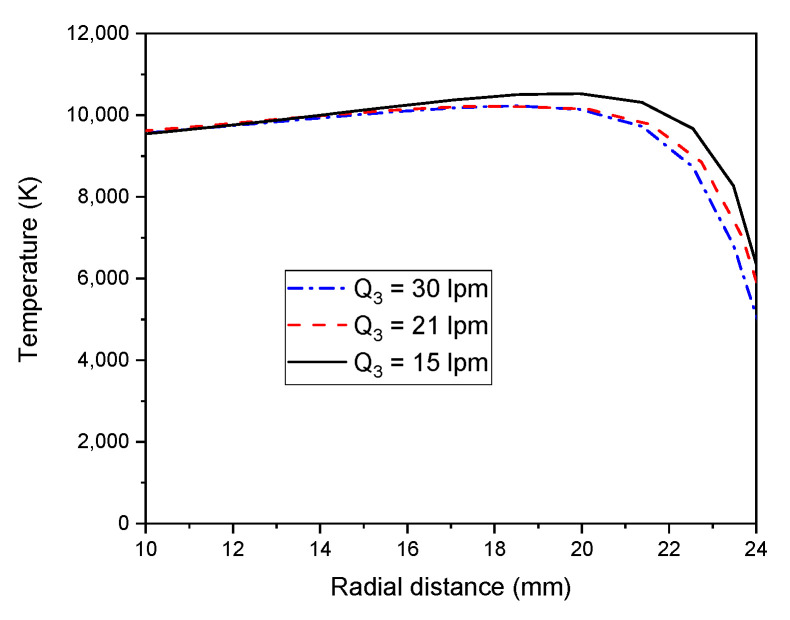
Radial temperature field distribution (z = 92 mm) at a different gas flow rate (zoomed view from 10 to 24 mm).

The temperature changes of an ICP torch were investigated as a function of the sheath gas flow rate (see, Figure 10). The plasma temperature decreases near the torch wall for an elevated flow rate of sheath gas. This could be assigned to a wider diffusion of sheath gas into the plasma resulting in high energy loss. Consequently, a decline in the peak temperature observed is associated with a diminution of the skin depth defined as signal penetration distance. At a high sheath gas flow rate, the maximum region of electrical conductivity moves towards the wall, due to the skin effect [59]. 

**Figure 11:**
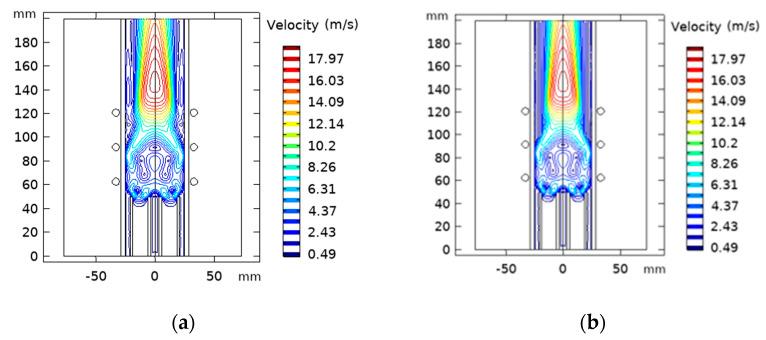
Contours of flow fields for different sheath gas flow rate Q_1_ = 15 lpm (**a**), Q_2_ = 21 lpm (**b**).

In Figure 11, the velocity flow field shows a circulating region near the coil region at a lower flow rate. This is due to the radial Lorentz force generated by the induction electromagnetic fields in the plasma that pinch the plasma flow field. However, this recirculating region disappears at a higher flow rate, since the Lorentzian pinch effect is overridden by inertia force. 

**Figure 12:**
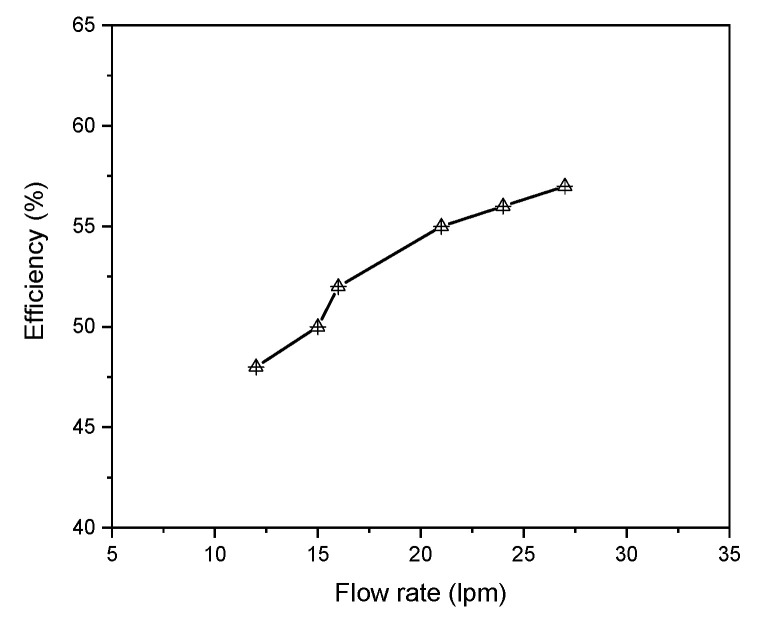
Efficiency as a function of sheath gas flow rate.

As shown in Figure 12, the efficiency rises as the sheath gas flow rate augments. The torch coupling efficiency is improved since a higher gas flow removes more heat from the torch channel.

**Figure 13:**
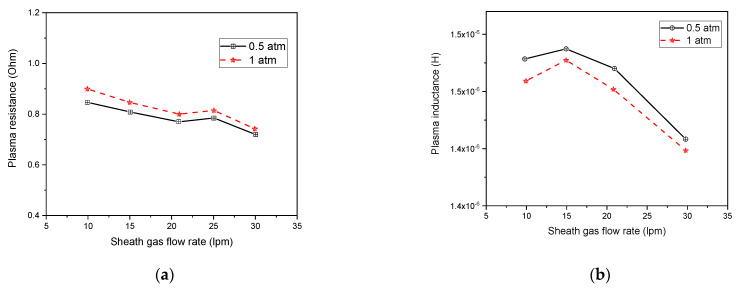
Plasma resistance (**a**) and inductance (**b**) with varying sheath gas flow rate at different pressures.

Figure 13a illustrates the variation of plasma resistance as a function of the sheath gas flow rate. Plasma resistance diminishes with increasing sheath gas flow rate due to the drop of the plasma volume contrary to its diameter, which remains unchanged.

Figure 13b demonstrates the drop of the torch inductance while rising the sheath gas flow rate. Indeed, a higher gas flow rate results in an important axial temperature value at the upstream coil location, which increases electrical conductivity and simultaneously decreases torch inductance. Hence, the plasma resistance rises while the corresponding inductance decreases as the pressure augments.

According to the local thermodynamic equilibrium property of ICP thermal plasma, the thermodynamic properties vary with temperature and pressure. A high-pressure operating regime is characterized by lower variation of physical properties with pressure as compared to low-pressure operating regime [60]. Thus, the influence of the pressure variation on electric characteristic plasma is relatively small.

#### 4.3.3. Nobel Sheath Gas Composition Effect on the Plasma Torch

Insulating the sidewall with a higher ionized sheath gas than Argon, like Helium and Hydrogen, reduces the energy lost through the sidewall while moving the plasma heated zone towards the center of the torch [61]. However, limited Helium quantity must be used to avoid turning off the flame and inefficiency in material processing. 

**Figure 14:**
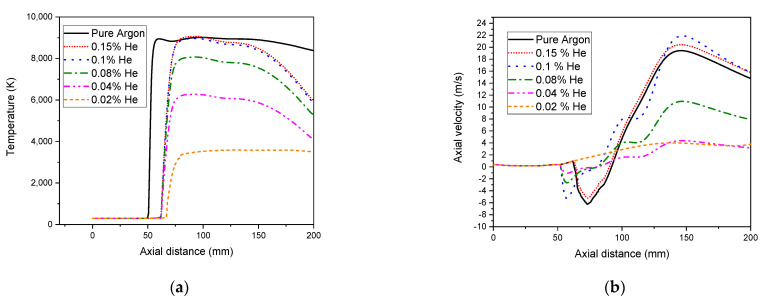
Effect of sheath gas addition on axial temperature (**a**) and axial velocity distributions (r = 0) (**b**).

In Figure 14, adding Helium to Argon, a temperature decrease is observed, where the plasma is cooled down (Figure 14a), the axial velocity is reduced, and the circulating flow is eliminated (Figure 14b). 

This phenomenon could be assigned to the smaller atomic mass of Helium compared to Argon. Then, Helium is more difficult to ionize than the operating gas Argon. So, using Helium/Agon improves the ability of the sheath gas to transfer heat and allows for the shift of the heated plasma zone away from the torch side wall. Consequently, both drops of velocity and temperature observed here are originated from the collisional cooling through the plasma. Furthermore, the striking effect of buffer gas addition eliminates the circulating flow that consumes energy and improves the torch efficiency. This becomes more pronounced while using 0.02% of Helium, resulting in the least energy loss. Above this value, the temperature is reduced for comparing to a pure Argon case. However, the velocity rises even more than in a pure Argon case. More energy is lost to the water cooling in the side wall of the torch, and the plasma can be extinguished by excess of Helium.

#### 4.3.4. Effect of Swirl Flow

**Figure 15:**
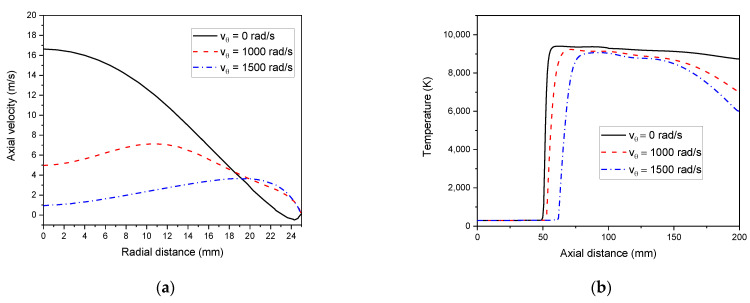
Effect of swirl flow on axial velocity (z = 92 mm) (**a**) and axial temperature (r = 0) (**b**).

Figure 15a represents the swirl flow effect on axial velocity. The swirl flow diminishes the flow of axial velocity and thereby raises the material particles residence time. This mainly favors nanoparticle synthesis [62]. Indeed, the swirl flow along the symmetry axis reduces the axial velocity, but contrarily rises the radial velocity to the torch wall side. So, as the swirl flow velocity increases, the tendency of flow separation decreases, and the vortex size decreases.

The important convection heat transfer existing along the radial direction into the wall highly reduces the temperature in the outlet (see, Figure 15b). Consequently, the wall heat loss increases, and the torch efficiency decreases. Thus, sheath swirl flow plays a major role in developing a free vortex that stabilizes plasma flow [63]. 

### 4.4. Effect of Geometry Torch

#### 4.4.1. Variation of Coil Spacing

Coil spacing “L_c_” was simulated at three different values in an ICP torch with three coils, including L_c_ = 14.5 mm, 29 mm, and 39 mm to measure the effect of coil spacing on the fluid flow. Coils are placed at 63 mm from the inlet for all variations of L_c_.

**Figure 16:**
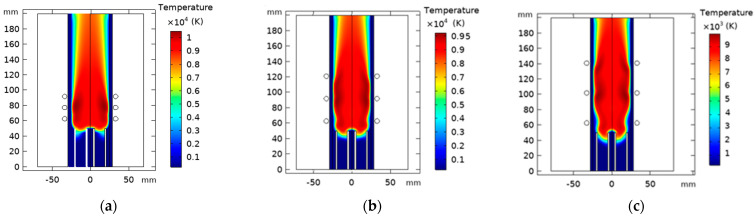
Temperature fields with varying turns coil spacing L_c_ = 14.5 mm (**a**), 29 mm (**b**), and 39 mm (**c**).

Figure 16 illustrates the temperature distribution of the ICP torch at various coil spacing. It appears that as we enlarge the separation distance between the coil, the maximum temperature decreases. Herein, the torch becomes unable to form a continuous area of high temperature. However, coils with a shorter spacing generate high local temperatures closer to the centerline. Near the outer wall, the temperature was slightly higher, forming a high-temperature ring-like area in the ICP torch when the temperature field stabilized [64]. 

**Figure 17:**
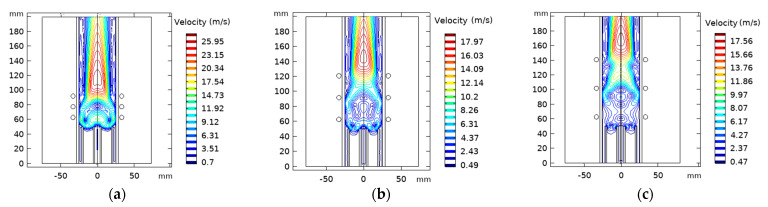
Velocity flow fields contours with varying turns coil spacing L_c_ = 14.5 mm (**a**), 29 mm (**b**), and 39 mm (**c**).

The velocity distribution at different coil separation distances is given in Figure 17. A larger recirculation region appears in the last coil edge when the turn coil distance is 14.5 mm and becomes smaller as the turns get further apart.

**Figure 18:**
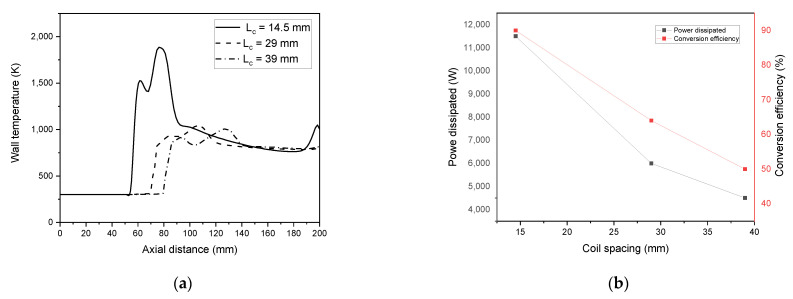
Wall temperature (**a**) and conversion efficiency and dissipated power (**b**) dependency on the change of the coil turns spacing.

Figure 18a represents the wall temperature tendency. The wall temperature is reduced as the coil’s separation distance augments. The spacing must be greater than L_c_ = 14.5 mm to avoid reaching the melting point temperature of the quartz tube that damages the torch wall (melting point tube is 1683 K). The total dissipated power decreases, ranging from 11.6 kW to 4.5 kW simultaneously with the conversion efficiency that drops to 42% with enlarging the spacing of the turns (see, Figure 18b).

#### 4.4.2. Effect of Turns Coil Number Variation

**Figure 19:**
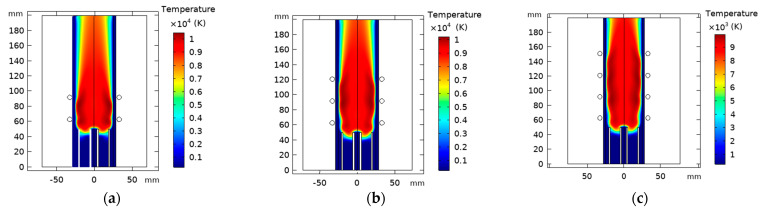
Temperature distribution with varying turn coil number, N_c_ = 2 (**a**), 3 (**b**), and 4 (**c**).

Herein, we admitted the same torch geometry characteristics. On the other hand, we fixed the separation distance from the inlet to the first coil to 63 mm, where we will vary the number of the coils “N_c_” from 2 to 4 with a constant spacing equal to 29 mm. Figure 19 represents the temperature distribution dependency on the coil turn numbers; there is an apparent increase in core temperature as the coil turns number rises.

**Figure 20:**
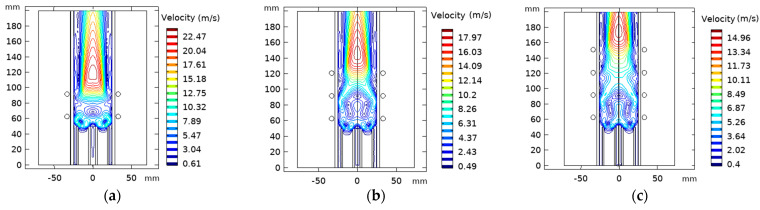
Velocity flow fields contours with varying turn coil number, N_c_ = 2 (**a**), 3 (**b**), and 4 (**c**).

The velocity flow field highly depends on the turns of the coil, as shown in Figure 20. The high-velocity areas remain independent of the change of the coil number, which is always positioned at the end of the last coil. The main vortex was observed at the inlet for the different studied cases. This is assigned to the high pressure and strong axial body force in the inlet. The second vortex appearing at the end of the last coil is influenced by the radial body force moving towards the wall.

**Figure 21:**
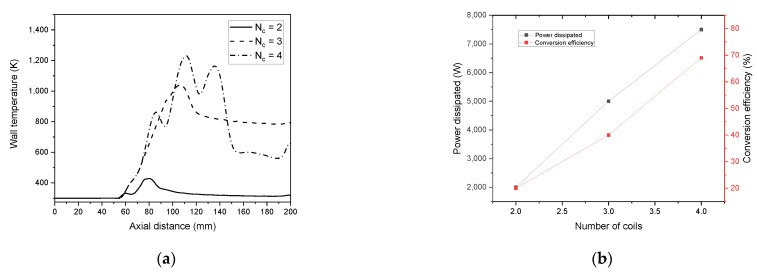
Wall temperature (**a**) and dissipated power and conversion efficiency (**b**) as a function of the separation distance between the turn of the coil.

The wall temperature profile for N_c_ = 2, 3, 4 is found to be equal to 428 K, 1039 K, and 1230 K, respectively (see, Figure 21a). N_c_ equal to 2 and 3 is suitable for wall temperature below the melting point of a quartz tube (≈1683 K). 

On the other hand, N_c_ equal to 4 could be used only if water cooling was associated with the quartz wall that reduces the temperature. Conversion efficiency increases from 25% to 68% as the coil number augments (see, Figure 21b).

## 5. Conclusions

In this work, the ICP torch was investigated. A practical focus was addressed on the condition performance, thus ensuring better optimization of the plasma characteristics. The major role of the efficient distribution of energy in the torch was highlighted. The numerical results are in good agreement with previous experimental measurements. A rise in the electrical conductivity of the plasma associated with a skin depth drop was observed as the power level increased. These results were deduced through the temperature peak shift from the centerline to the wall direction. Consequently, plasma resistance increases while plasma inductance decreases with rising RF power. High induction frequencies were found to be more efficient in transmitting power to the plasma. As the sheath gas flow rate increases, while the power remains constant, the plasma heat is transferred effectively to the sheath gas. The striking effect of the buffer layer addition shows that the 0.02% Helium incorporation to Argon gas makes the torch more efficient. The vortex formation could be diminished by the swirl sheath gas. This raises the wall heat transfer and decreases the temperature along the central line. We found that the increase in the coil spacing decreases: the wall temperature, the dissipated power, and the efficiency. Particularly, LC equal to 14.5 mm is not recommended experimentally because the wall temperature attains the melting point of quartz. On other the other hand, the number of coils equal to 2 or 3 is found practically suitable, contrary to Nc equal 4 which requires a water colling. The present model ensures an enhancement of energy efficiency of inductively coupled plasma torches, which can be accomplished at a higher power and frequency, by increasing mass flow rate, an addition of 0.02% helium to Argon sheath gas with swirl flow, small coil spacing, and having a number of coils equal to 2 or 3. 

## Figures and Tables

**Table 1:** Operating conditions and geometry dimension of ICP torch.

Dimensions	Value
Nozzle radius r_1_	3.7 mm
Nozzle radius r_2_	18.8 mm
Inner torch wall radius r_w_	25 mm
Thickness inner tube d	2 mm
Radius to center coil r_c_	33 mm
Axial position of lower coil z_1_	63 mm
Coil length z_c_	58 mm
Reactor length z_3_	200 mm
Wall thickness δ_w_	3.5 mm
Voltage waveform	Sinusoidal
Gas	Argon
Coil turn number N	3.0 turns
Ambient temperature T	300.0 K
Coil excitation power P	15.0 kW
Coil frequency f	3 MHz
Operational pressure p	1.0 atm
Injected flow rate Q_1_, Q_2_, Q_3_	1.0, 3.0, 21.0 lpm

## Data Availability

Data are contained within the article.

## References

[1] Givelet L., Truffier-Boutry D., Noël L., Damlencourt J.F., Jitaru P., Guérin T. (2021). Optimization and application of an analytical approach for the characterization of TiO_2_ nanoparticles in food additives and pharmaceuticals by single particle inductively coupled plasma-mass spectrometry. Talanta.

[2] Mozhayeva D., Engelhard C. (2020). A critical review of single particle inductively coupled plasma mass spectrometry—A step towards an ideal method for nanomaterial characterization. J. Anal. At. Spectrom..

[3] Isaguirre A.C., Moyano M.F., Gil R.A., Moglia M.M. (2020). A Novel and Simple Method for Elements Determination in Aerobiological Samples by Inductively Coupled Plasma Mass Spectrometry (ICP-MS) Analysis. Water Air Soil. Poll..

[4] Flores K., Turley R.S., Valdes C., Ye Y., Cantu J.A., Hernandez-Viezcas J.G., Parsons J.G., Gardea-Torresdey J.L. (2021). Environmental applications and recent innovations in single particle inductively coupled plasma mass spectrometry (SP-ICP-MS). Appl. Spectrosc. Rev..

[5] Tanvir E.M., Komarova T., Comino E., Sumner R., Whitfield K.M., Shaw P.N. (2021). Effects of storage conditions on the stability and distribution of clinical trace elements in whole blood and plasma: Application of ICP-MS. J. Trace Elem. Med. Bio..

[6] Brouwers E.E.M., Tibben M., Rosing H., Schellens J.H.M., Beijnen J.H. (2008). The application of inductively coupled plasma mass spectrometry in clinical pharmacological oncology research. Mass. Spectrom. Rev..

[7] Tan X., Liu M., He K. (2021). Study of Long-Term Determination Accuracy for REEs in Geological Samples by Inductively Coupled Plasma Quadrupole Mass Spectrometry. Molecules.

[8] Sun H., Yao Y., Loomis R.A., Buhro W.E. (2020). Methods for the ICP-OES Analysis of Semiconductor Materials Calynn Morrison. Chem. Mater..

[9] Lee J.J. (2005). Application of inductively coupled plasma to CVD and PVD. Surf Coat. Tech..

[10] Qin X.Z., Yang G., Cai F.P., Jiang B., Chen H., Tan C.H. (2019). Recovery and reuse of spent LiFePO4 batteries. J. New Mater. Electrochem. Syst..

[11] Gabbar H.A., Darda S.A., Damideh V., Hassen I., Aboughaly M., Lisi D. (2021). Comparative study of atmospheric pressure DC, RF, and microwave thermal plasma torches for waste to energy applications. Sustain. Energy Technol. Assessments..

[12] Isaac H. (2021). Study of Radio Frequency Inductively Coupled Thermal Plasma Torch (RF ICPT) for Radioactive Waste Treatment: Thermoplastics—Polyethylene and Polyvinyl Chloride (PVC). Master’s Thesis.

[13] Zhu B., Alavi S., Cheng C., Sun H., Zhao H., Kim K.S., Mostaghimi J., Zou Y. (2020). Fast and High-Throughput Synthesis of Medium- and High-Entropy Alloys Using Radio Frequency Inductively Coupled Plasma. Adv. Eng. Mater..

[14] Aldakheel R.K., Gondal M.A., Almessiere M.A., Rehman S., Nasr M.M., Alsalem Z., Khan F.A. (2021). Spectrochemical analysis using LIBS and ICP-OES techniques of herbal medicine (Tinnevelly Senna leaves) and its anti-cancerous/antibacterial applications. Arab. J. Chem..

[15] Wang D., Hao Z., Zhu X., Zhou F., Shu Y., He J. (2022). spheroidization of lithium niobate powder by radiofrequency inductively coupled plasma. Ceram Int..

[16] Racka-Szmidt K., Stonio B., Zelazko J., Filipiak M., Sochacki M. (2022). A Review: Inductively Coupled Plasma Reactive Ion Etching of Silicon Carbide. Materials.

[17] Mirek P., Alavi S., Mostaghimi J. (2021). A Novel Radio Frequency Inductively Coupled Plasma Torch for Material Processing. Plasma Chem. Plasma Process..

[18] Samal S., Tyc O., Cizek J., Klecka J., Lukác F., Molnárová O., De Prado E., Weiss Z., Kopecek J., Heller L. (2021). Fabrication of Thermal Plasma Sprayed NiTi Coatings Possessing Functional Properties. Coatings.

[19] Zhang X., Hayashida R., Tanaka M., Watanabe T. (2020). Synthesis of carbon-coated silicon nanoparticles by induction thermal plasma for lithium ion battery. Powder Technol..

[20] Thompson M., Walsh J.N. (1989). Handbook of Inductively Coupled Plasma Spectrometry.

[21] Boulos M.I. (2016). The role of transport phenomena and modelling in the development of thermal plasma technology. Plasma Chem. Plasma Process..

[22] Yu N., Yang Y., Jourdain R., Gourma M., Bennett A., Fang F. (2020). Design and optimization of plasma jet nozzles based on computational fluid dynamics. Int. J. Adv. Manuf. Tech..

[23] Yu N., Jourdain R., Gourma M., Xu F., Bennett A., Fang F. (2021). Power Dissipation of an Inductively Coupled Plasma Torch under E Mode Dominated Regime. Micromachines.

[24] Boulos M.I. (1985). The inductively coupled R.F. (radiofrequency) plasma. Pure Appl. Chem..

[25] Punjabi S.B., Das T.K., Joshi N.K., Mangalvedekar H.A., Lande B.K., Das A.K. (2010). The effect of various coil parameters on ICP torch simulation. J. Phys. Conf. Ser..

[26] Lindner H., Bogaerts A. (2011). Multi-element model for the simulation of inductively coupled plasmas: Effects of Helium addition to the central gas stream. Spectrochim. Acta Part B At. Spectrosc..

[27] Merkhouf A., Boulo M.I. (2000). Distributed energy analysis for an integrated radio frequency induction plasma system. J. Phys. D Appl. Phys..

[28] Merkhouf A., Boulos M.I. (1998). Integrated model for the radio frequency induction plasma torch and power supply system. Plasma Sources Sci. Technol..

[29] Xue S., Proulx P., Boulos M.I. (2001). Extended-field electromagnetic model for inductively coupled plasma. J. Phys. D Appl. Phys..

[30] Cai M., Montaser A., Mostaghimi J. (1993). Computer simulation of atmospheric pressure Helium inductively coupled plasma discharges. Spectrochim. Acta Part B At. Spectrosc..

[31] Bernardi D., Colombo V., Ghedini E., Mentrelli A. (2003). Comparison of different techniques for the FLUENT based treatment of the electromagnetic field in inductively coupled plasma torches. Eur. Phys. J. D.

[32] Fouladgar J., Chentouf A. (1993). The calculation of the impedance of an induction plasma installation by a hybrid finite-element boundary-element method. IEEE Trans. Magn..

[33] Ye R., Proulx P., Boulos M.I. (1999). Turbulence phenomena in the radio frequency induction plasma torch. Int. J. Heat Mass Tran..

[34] (2019). COMSOL AB. COMSOL Multiphysics V5.4. Multiphysics Particle Tracing Module User’s Guide; COMSOL AB: Stockholm, Sweden. https://doc.comsol.com/5.3/doc/com.comsol.help.particle/ParticleTracingModuleUsersGuide.pdf.

[35] Bolot R., Coddet C., Schreuders C., Leparoux M., Siegmann S. (2007). Modelling of an Inductively Coupled Plasma for the Synthesis of Nanoparticles. Journal of Thermal Spray Technology. J. Spray Techn..

[36] Shigeta M., Sato T., Nishiyama H. (2004). computational simulation of the particle laden RF inductively coupled plasma with seeded potassium. Int. J. Heat Mass Tran..

[37] Mostaghimi J., Proulx P., Boulos M.I. (1985). Parametric study of flow and temperature fields in an inductively coupled RF Plasma Torch. J. Plasma Chem. Plasma.

[38] Yang J.G., Yoon N.S., Kim B.C., Choi J.H., Lee G.S., Hwang S.M. (1999). Power Absorption Characteristics of an Inductively Coupled Plasma Discharge. IEEE Trans. Plasma Sci..

[39] Eizaguirre S., Gehring T., Denk F., Simon C., Kling R. Argon ICP plasma torch at atmospheric pressure driven by a SiC based resonant converter operating in MHz range. Proceedings of the International Exhibition and Conference for Power Electronics, Intelligent Motion, Renewable Energy and Energy Management (PCIM Europe Digital Days 2020).

[40] Punjabi S.B., Barve D.N., Joshi N.K., Das A.K., Kothari D.C., Ganguli A.A., Sahasrabhude S.N., Joshi J.B. (2019). Computational Fluid Dynamics (CFD) Simulations and Experimental Measurements in an Inductively- Coupled Plasma Generator Operating at Atmospheric Pressure: Performance Analysis and Parametric Study. Processes.

[41] Turkoz E. (2014). Numerical Model for Axisymmetric Inductively Coupled Plasma (ICP) in Radiofrequency (RF) Ion Thrusters. Master’s Thesis.

[42] Kim J., Mostaghimi J., Iravani R. (1997). Performance Analysis of a Radio-Frequency Induction Plasma Generator Using Nonlinear State-Space Approach. IEEE Trans. Plasma Sci..

[43] Chentouf A., Fouladgar J., Develey G. (1995). A simplified method for calculation of the impedance of an induction plasma. IEEE Trans. Magn..

[44] Grover F.W. (1946). Inductance calculations. Circuits Systems..

[45] Boulos M.I., Fauchais P., Pfender E. (1994). Thermal Plasmas: Fundamentals and Applications.

[46] Dodt D.H. (2009). Determination of the Electron Energy Distribution Function of a Low Temperature Plasma from Optical Emission Spectroscopy. PhD’s Thesis.

[47] Bernardi D., Colombo V., Ghedini E., Mentrelli A. (2003). Three-dimensional Effects in the Modelling of ICPTs—Part I: Fluid Dynamics and Electromagnetics. Eur. Phys. J. D.

[48] (2015). COMSOL AB. COMSOL Multiphysics® v. 5.4; COMSOL AB: Stockholm, Sweden. https://www.comsol.com/.

[49] Xie Z., Pavlidis D., Salinas P., Pain C., Matar O. (2020). A control volume finite element method for three-dimensional three-phase flows. Int. J. Numer. Meth. Fluids..

[50] Punjabi S.B., Sahasrabuddhe S.N., Ghorui S., Joshi N.K., Das A.K., Kothari D.C., Ganguli A.A., Joshi J.B. (2014). Flow and temperature patterns in the coil region of Inductively Coupled Plasma Reactor: Experimental measurements and CFD simulations. AIChE J..

[51] Lesinski J., Gagne R., Boulos M.I. (1981). Gas and Particle Velocity Measurements in an Induction Plasma. Technical Report.

[52] Punjabi S.B., Joshi N.K., Mangalvedekar H.A., Lande B.K., Das A.K., Kothari D.C. (2012). A comprehensive study of different gases in inductively coupled plasma torch operating at one atmosphere. Phys. Plasmas..

[53] Mostaghimi J., Boulos M.I. (1989). Two-Dimensional Electromagnetic Field Effects in Induction Plasma Modelling. Plasma Chem. Plasma Process..

[54] Shigeta M. (2012). Time-dependent 3D simulation of an Argon RF inductively coupled thermal plasma. Plasma Sources Sci. Technol..

[55] Khalili A., Sadat Kiai S.M., Mahdian H. (2016). Collisional cooling in an inductively coupled plasma torch. J. Phys. Astron. JOPA.

[56] Alavi S., Mostaghimi J. (2019). A Novel ICP Torch with Conical Geometry. Plasma Chem. Plasma Process..

[57] Grobler N.J.M., Bissett H., Puts G.J., Crouse P.L. Finite-element analysis of the effect of sheath-gas composition in an inductively coupled plasma. Proceedings of the Conference of the South African Advanced Materials Initiative (CoSAAMI-2018).

[58] Carcione J.M. (2006). A spectral numerical method for electromagnetic diffusion. Geophysics.

[59] Nishiyamay H., Muro Y., Kamiyama S. (1996). The control of gas temperature and velocity fields of a RF induction thermal plasma by injecting secondary gas. J. Phys. D Appl. Phys..

[60] Abeele D.V., Degrez G. (2004). Similarity analysis for the high-pressure inductively coupled plasma source. Plasma Sources Sci. Technol..

[61] Deng J., Zhang J., Zhang Q., Xu S. (2021). Effects of induction coil parameters of plasma torch on the distribution of temperature and flow fields. Alex. Eng. J..

[62] Elaissi S., Ben Gouider Trabelsi A., Alkallas F.H., Alrebdi T.A., Charrada K. (2022). Modeling of Advanced Silicon Nanomaterial Synthesis Approach: From Reactive Thermal Plasma Jet to Nanosized Particles. Nanomaterials.

[63] Xue S., Proulx P., Boulos M.I. (2003). Effect of the coil angle in an inductively coupled plasma torch: A novel two-dimensional model. Plasma Chem. Plasma Process..

[64] Kulacki F.A., Acharya S., Chudnovsky Y., Cotta R.M., Devireddy R., Dhir V.K., Pinar Mengüç M., Mostaghimi J., Vafai K. (2018). Handbook of Thermal Science and Engineering.

